# Changes in sex hormone levels after radical prostatectomy: Results of a longitudinal cohort study

**DOI:** 10.3892/ol.2013.1420

**Published:** 2013-06-21

**Authors:** MAURO GACCI, NICOLA TOSI, GIANNI VITTORI, ANDREA MINERVINI, GIOVANNI CORONA, TOMMASO CAI, ANNA MARIA MORELLI, LINDA VIGNOZZI, SERGIO SERNI, MARIO MAGGI, MARCO CARINI

**Affiliations:** 1 Department of Urology, University of Florence, Florence, Italy; 2 Endocrinology Unit, Maggiore-Bellaria Hospital, Bologna, Italy; 3 Department of Urology, Santa Chiara Regional Hospital, Trento, Italy; 4 Department of Clinical Physiopathology, University of Florence, Florence, Italy

**Keywords:** prostate neoplasms, radical prostatectomy, hypogonadism, serum testosterone, luteinizing hormone, follicle-stimulating hormone

## Abstract

The changes in testosterone and gonadotropin levels in patients who have undergone radical prostatectomy (RP) for clinically localized prostate cancer (PCa) remain unclear. The aim of the present study was to prospectively evaluate the changes in serum testosterone (Te), luteinizing hormone (LH) and follicle-stimulating hormone (FSH) levels in the early months after RP for PCa and the correlation between these hormones at various follow-up times. A total of 100 male patients with clinically localized PCa were consecutively included in the study. The serum levels of Te, LH and FSH were measured prior to RP (baseline) and at 1 and 3 months post-operatively. Changes in the levels of Te, LH and FSH between the baseline and at 1 and 3 months after RP were analyzed with paired sample t-tests. The correlations between LH and Te levels at the various follow-up times were evaluated with a Spearman’s rank correlation coefficient. At 1 month subsequent to RP, the Te levels were significantly decreased (baseline vs. 1 month, P=0.021) and subsequently recovered to the pre-operative value at 3 months (baseline vs. 3 months, P=0.372). The mean Te level at baseline was 15.3 nmol/l, while at 1 and 3 months it was 13.8 and 14.4 nmol/l, respectively. By contrast, the levels of LH and FSH were significantly increased at 1 and 3 months post-surgery, compared with the baseline value (baseline vs. 1 or 3 months, P<0.0001). The pre-operative correlation between LH and Te was lost 1 month after RP and recovered after 3 months. Notably, the LH level at 1 month was markedly correlated with the Te levels recorded after 3 months. In the present study, patients developed compensated hypergonadotropic hypogonadism 3 months after RP.

## Introduction

Several studies have demonstrated that erectile dysfunction and lower urinary tract symptoms due to prostatic disorders may share a common pathophysiological pathway (1,2). The serum level of testosterone (Te) may affect sexual function in males treated by radical prostatectomy (RP) for clinically localized prostate cancer (PCa) (3). It has been suggested that Te may also affect urinary continence and increase bladder compliance following RP, with a relaxing effect in smooth muscle bladder cells through the nitric oxide synthase/nitric oxide pathway (4,5). Moreover, certain authors have reported a significant correlation between Te levels and adverse pathological features and biochemical recurrence, even though the prognostic value of pre-operative Te remains unclear (6–7). Few preliminary studies have evaluated the changes in the levels of Te and gonadotropins following RP, and these have produced conflicting results (9–12). In addition, little is known concerning the possible effect of PCa cells on the hypothalamic-pituitary hormone axis, although certain authors have suggested that patients with PCa showed lower gonadotropin levels compared with similarly aged males without PCa (12). Furthermore, there is no data with regard to changes in sex hormones in the early post-operative period following RP. The aim of the present study was to prospectively evaluate the changes in the serum levels of Te, luteinizing hormone (LH) and follicle-stimulating hormone (FSH) within the first 3 months after RP for clinically localized PCa and to analyze the correlation between LH and Te at various follow-up times.

## Materials and methods

### Study design

A total of 100 male patients with clinically localized PCa were consecutively included in the study, from February to July 2010. The levels of Te, LH and FSH were assessed pre-operatively and at 1 and 3 months post-operatively. This study was approved by the ethics committee of the University of Florence. Informed consent was obtained from all patients. This study was planned as a longitudinal cohort study.

### Patients

A total of 100 male patients with newly diagnosed, biopsy proven, clinically localized PCa [prostate-specific antigen (PSA) <10 mg/ml, clinical stage <T3, negative computed tomography (CT) scan or bone scintigraphy] prior to RP were included in the study. Subjects with a known history of endocrinological diseases, including hypogonadism, previous Te substitutive therapy and/or hormonal manipulation (including 5α-reductase inhibitors, androgen receptor blockers and LH-releasing hormone analogues), were excluded from the study. The adjunctive exclusion criteria were a classification of Eastern Cooperative Oncology Group (ECOG) grade >1, previous radiation therapy and chronic ingestion of alcohol or other drugs, including steroids, barbiturates, spironolactone and cimetidine, that may have interfered with the serum hormone levels.

### Clinical and serological data collection

The clinical variables registered for each patient included age at surgery, corporal features [body mass index(BMI), weight and height] and sex hormone profiles, including total Te, LH and FSH. Serum samples were collected by venipuncture between 8:00 and 11:00 a.m., 7 to 14 days prior to RP and at the 1- and 3-month follow-ups. The serum levels of Te, LH and FSH were quantified using an electrochemoluminescence method (Modular Roche, Milan, Italy) at the laboratories of the Sexual Medicine Outpatient Clinic, University of Florence, Italy.

### Surgical procedures

The surgical indications were in accordance with the European Association of Urology guidelines for clinically localized PCa (13). RP was performed with an open retropubic antegrade approach, according to our previously described surgical procedures (14). All surgical procedures were performed at the Department of Urology, University of Florence, Florence, Italy, by the same 3 surgeons (M.G., M.C. and S.S.). Each patient provided written informed consent prior to their surgical procedure.

### Statistical analysis

The Kolmogorov-Smirnov test was used to test the distribution of each parameter. Data are expressed as the median (quartiles) when not normally distributed and as the mean ± SD when normally distributed. The distributions of LH and FSH were normalized through logarithmic transformations: logarithmically transformed LH and FSH were expressed as logLH and logFSH, respectively.

Comparisons between the serum hormone levels at baseline and the levels at 1 and 3 months after RP were performed with paired t-tests for normally distributed parameters. The same test was applied to the logarithmically transformed data for LH and FSH. The correlation between the logarithmically transformed LH and Te values at each visit was calculated using Pearson’s correlation coefficient. All statistical tests were performed using SPSS version 17.0 statistical software (SPSS, Inc., Chicago, IL, USA).

## Results

### Pre-operative data

The baseline patient characteristics are shown in Table I. Subsequent to 3 months, complete hormonal data (baseline, 1 and 3 months) were available for 92/100 patients. In total, 4 patients voluntarily withdrew from the study and were excluded from the results, while 4 were not analyzed due to adjuvant radiation and/or hormonal treatment for persistent PCa (R+, N+) or as they were lost to follow-up.

### Changes in sex hormone levels following RP

**Figure 1.**
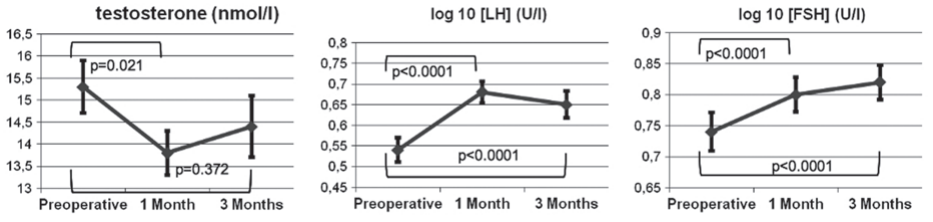
Comparisons of the serum hormone levels at baseline with those at 1 and 3 months from 92 PCa patients with complete data. Paired t-tests were used for Te (normally distributed, expressed as nmol/l) and logarithmically transformed LH and FSH (not normally distributed, expressed as U/l). Data are expressed as the mean ± standard error of the mean. LH, luteinizing hormone; FSH, follicle-stimulating hormone; Te, testosterone; PCa, prostate cancer.

**Figure 2.**
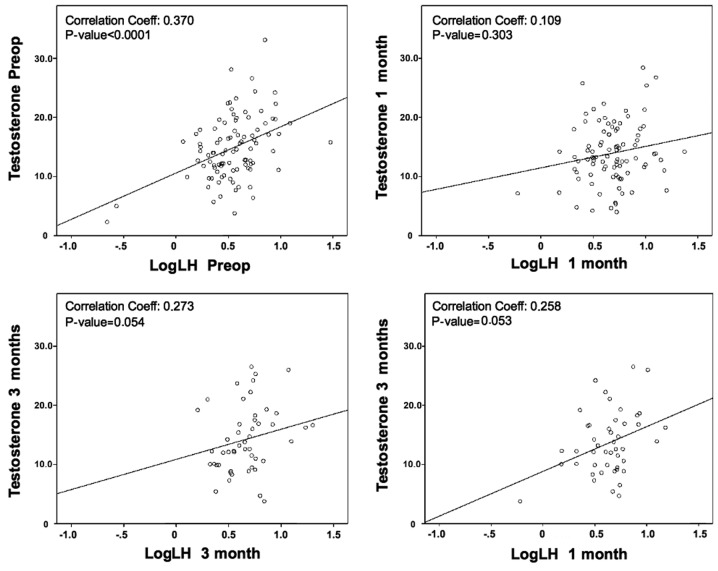
Correlation analysis (Pearson’s correlation coefficient) between testosterone and logarithmically transformed LH values at each visit of the study, of the 92 PCa patients with complete data. LH, luteinizing hormone; Pre-op, pre-operative; PCa, prostate cancer.

The results of the laboratory analyses of Te, logLH and logFSH are shown in Fig. 1. A significant reduction in Te levels was observed 1 month after RP, as compared with the baseline (baseline vs. 1 month, 15.3 vs. 13.8 nmol/l; P=0.021), with an increase at 3 months that nearly reached the pre-operative baseline value (baseline vs. 3 months, 15.3 vs. 14.4 nmol/l; P=0.372). In contrast, the logLH level was significantly increased at 1 and 3 months post-surgery, compared with the baseline (baseline vs. 1 month logLH, 0.54 vs. 0.68 mIU/ml; P<0.0001; and baseline vs. 3 months logLH, 0.54 vs. 0.65 mIU/ml; P<0.0001). Furthermore, the logFSH level was significantly increased at 1 month after RP (baseline vs. 1 month logFSH, 0.74 vs. 0.80 mIU/ml; P<0.0001) and reached the highest value at the 3-month follow-up (baseline vs. 3 months logFSH, 0.74 vs. 0.82 mIU/ml; P<0.0001). The correlation between the LH and Te serum levels was then evaluated at various follow-up times (Fig. 2). A marked positive correlation was observed between Te and LH pre-operatively (pre-operative logLH-Te, r=0.370; P<0.0001). This correlation was not apparent 1 month after RP (one month logLH-Te, r=0.109; P=0.303). However, the LH level measured at 1 month was markedly correlated with the Te level at 3 months (one month logLH-3 month Te, r=0.258; P=0,053). At 3 months post-surgery, the correlation between Te and LH was completely recovered (three month logLH-Te, r=0.273; P=0.054).

## Discussion

In the early months after RP, the present study observed a significant decline and subsequent recovery of Te, associated with increased LH and FSH, which persisted at higher levels at 3 months compared with the pre-operative value. Therefore, the removal of the whole prostate gland was followed by the development of compensated hypergonadotropic hypogonadism. To date, only a few studies have evaluated the changes in sex hormone levels following prostatic surgery, and little is known with regard to the effect of healthy prostatic tissue or PCa on the hypothalamic-pituitary hormone axis (15).

Certain studies have shown that there are no significant changes in these hormones following surgical treatment for benign prostatic hyperplasia (BPH) (12,16,17). Further investigations are required, but on the basis of these studies it appears that BPH does not significantly affect the hypothalamic-pituitary axis. In contrast, other studies have previously presented changes in the levels of sex hormones after RP for PCa, but there is no consensus with regard to the existence and degree of these changes. Olsson *et al* reported a significant increase in LH and FSH levels in 55 males following RP in a pilot study (10). These findings have been supported by Madersbacher *et al*, who observed that LH and FSH were increased by 71 and 63%, respectively, in 49 patients 12 months after RP, without any evident changes to the Te level (12). In a slightly different manner, Miller *et al* showed a significant increase in Te, as well as LH and FSH, in 63 patients 1 year after RP, when compared with the pre-operative value (9). There are two main differences between the present and previous studies. First, the first post-operative evaluation was subsequent to only 1 month, whereas in the previous studies, it was at 3 or 6 months. This enabled us to make the new observation, not reported by previous studies, that Te was transiently decreased 1 month after RP. The second difference was the correlation analysis with LH. The decrease in Te levels at 1 month was unassociated with LH, while at 3 months, the Te level recovered the correlation with LH, as well as its baseline value. A possible explanation for these data is that the surgical intervention caused a reduction in the testicular production of Te, which stimulated the increased production of gonadotropins by negative feedback, thus normalizing the Te level after a few weeks. The reduction in Te levels that was identified at the 1-month follow-up may be a significant etiological factor of the increased gonadotropins, which have previously been observed even at 12 months after RP (9,10,12). In the present study, we were not able to dismiss other potential explanations, nor identify the cause of the reduced testicular production of Te following RP. However, we suggest that there may be numerous factors, including psychological and organic considerations. The psychological stress of facing a cancer diagnosis or the fear of surgery have already been considered as potential causes of the suppression of LH/FSH prior to RP (12). This may also cause a temporary reduction in testicular endocrine function. Organic factors, e.g., the ligature of Santorini’s venous plexus or of prostatic vascular pedicles and the Trendelenburg position, may theoretically cause transient ischemic or hypoxic damage to the testicles. Moreover, damage to the cavernous nerves may alter testicular function in humans, as has been reported in animal models (18). Following a bilateral cavernous neurotomy in rats, Vignozzi *et al* observed the onset of overt hypogonadism, characterized by reduced Te levels and testis function, including testis weight and number of Leydig cells, with an inadequate compensatory increase of LH (19). More detailed studies are required to completely investigate whether and how RP has an effect on the testicular production of Te, and if this is similar to the effect that is described after external beam radiation therapy (20).

Previous studies have presented various interpretations of the increased gonadotropin levels following RP, alternately attributing them to the removal of the PCa or healthy prostatic tissue, or to the surgical event itself. In the study by Madersbacher *et al,* the authors suggested that the hypothalamic-pituitary axis is inhibited in patients with PCa and that this inhibition is removed following RP. The authors presented evidence for a possible direct inhibitory effect of tumor cells, as there was a correlation between higher Gleason scores and lower pre-operative Te, and significantly lower LH and FSH levels were observed in the PCa group compared with the BPH group (12). However, Miller *et al* suggested that it was the healthy prostatic tissue, producing dihydrotestosterone (DHT) and inhibin-B, that caused the pre-operative inhibition of the hypothalamic-pituitary axis (9). Furthermore, DHT induces negative feedback for LH and FSH, and decreases in its levels in serum and urine following RP have also been observed in other studies (10,12). By contrast, Olsson *et al* did not report any changes to the serum inhibin-B levels in 44 patients following RP, nor any difference between the PCa and BPH patient groups (10). The present study does not disagree with any previous justifications, although it does provide a new observation (the early reduction of Te) which may present a new interpretation. Further studies are required to confirm whether RP affects testicular production of Te. In the present study, this premise is suggested by the levels of LH and FSH, which remained high at 3 months compared with the baseline value, despite a normal Te value. This premise is also supported by the correlation analysis between Te and LH, which showed that the correlation was lost at 1 month and recovered at 3 months. This explanation would also justify the increased gonadotropin levels observed with the normal Te levels in previous studies at 6 and 12 months following RP (9,12). In addition to possessing significance in endocrinological terms, the fluctuation of the Te levels in the first 3 months following RP may be important from a clinical perspective, since this period of time has critical relevance in the recovery of urinary continence and potency (21), and the resultant Te levels are significantly involved in the recovery of these functions (4,5). Although the present study did not record data on the functional recovery of these patients, we suggest that future investigations into the correlation between functional recovery and sex hormones should focus on the first months after prostatectomy. In our opinion, the strengths of the present study are inherent in its prospective nature and the high significance of the statistical results; conversely, the limits include the lack of sex hormone-binding globulin (SHBG) and estrogens results in the analysis, which were excluded due to their cost. However, not all previous studies on this issue have included SHBG and estrogens in the evaluation, and no remarkable adjunctive outcomes have resulted from those that did (9–12). In conclusion, in the present series, RP induced an early significant decline in the Te serum levels and a significant increase in the LH and FSH levels. At 3 months after RP, the full recovery of the Te level, with concomitant high levels of gonadotropins, appears to delineate the features of compensated hypergonadotropic hypogonadism. Studies evaluating the effect of these hormonal changes on continence and potency are currently in progress.

## Figures and Tables

**Table I.** Pre-operative clinical and hormonal characteristics of 100 male patients with PCa.

Characteristic	Value
Pre-operative clinical data	
Age	
Mean ± SD (years)	64.8±6.5
Range (years)	48–75
<65 (n)	52
≥65 (n)	48
BMI (kg/m^2^)	
Mean ± SD	25.8±3.1
Range	19.5–34.6
PSA (ng/ml)	
Mean ± SD	7.3±4.0
0–2.4	6
2.5–3.9	8
4–10	69
10.1–19.9	17
Pathological outcome	
Pathological Stage (n)	
pT2	54
pT3	46
pT4	0
Gleason Score of surgical specimens (n)	
<6	0
6	55
7	29
8–10	16
Lymph nodes status (n)	
N0	98
N+	2
Surgical margins (n)	
Positive	2
Negative	98
Hormone levels	
Testosterone (nmol/ml)	
Mean ± SD	15.3±5.9
Range	5.7–37.8
LH (mIU/ml)	
Median	3.5
Quartiles	2.5–3.5–5.1
FSH (mIU/ml)	
Median	5.0
Quartiles	3.3–5.0–8.1

Values are expressed as the mean ± SD when normally distributed and as median (quartiles) when not normally distributed. LH, luteinizing hormone; FSH, follicle-stimulating hormone; PSA, prostate-specific antigen; BMI, body mass index; PCa, prostate cancer.
